# Intraoperative tranexamic acid use in elderly patients with intertrochanteric fractures: impact on early postoperative inflammation and clinical outcomes

**DOI:** 10.3389/fphys.2026.1801500

**Published:** 2026-03-27

**Authors:** Leiming Jiang, Chaoqun Feng, Kaipeng Chen, Hongjie Yang, Zhen Chen

**Affiliations:** 1 Department of Orthopedics, Hospital of Chengdu University of Traditional Chinese Medicine, Chengdu, Sichuan, China; 2 Chengdu University of Traditional Chinese Medicine, Chengdu, Sichuan, China

**Keywords:** elderly, hip fractures, inflammation, intertrochanteric fractures, tranexamic acid

## Abstract

**Background and purpose:**

Tranexamic acid (TXA) demonstrates anti-inflammatory effects during surgery. However, the clinical value of the TXA in intertrochanteric fractures patients has not been further investigated. This study aimed to investigate the effects of intraoperative tranexamic acid (TXA) administration on early postoperative inflammatory markers and clinical outcomes in elderly patients with intertrochanteric fractures.

**Patients and methods:**

A total of 136 patients (59 males and 77 females) aged 62–96 years were enrolled. Patients were divided into two groups: 68 received TXA [TXA group, intravenous drip (15 mg/kg)] and 68 did not receive TXA (control group). We compared surgical duration, total blood loss (TBL), hidden blood loss (HBL), transfusion rates, incidence of deep vein thrombosis (DVT), length of hospital stay, serum interleukin-6 (IL-6) levels on postoperative days 1, 3, 5, and 7, and visual analog scale (VAS) scores.

**Results:**

The TXA group exhibited significantly lower TBL (981.74 ± 451.14 mL vs. 1206.27 ± 408.22 mL, p = 0.002), HBL (890.94 ± 409.99 mL vs. 1104.39 ± 412.06 mL, p = 0.003), and transfusion rates (32.3% vs. 51.4%, p = 0.037) compared with the control group. Serum IL-6 levels and VAS scores were significantly lower in the TXA group on postoperative days 1 and 3 (p < 0.05). The complication rate within one year was also lower in the TXA group (26.4% vs. 48.5%, p = 0.032). No significant differences were observed between groups for surgical duration, DVT incidence, length of hospital stay, or one-year mortality (p > 0.05).

**Conclusion:**

Intraoperative TXA administration appears to effectively reduce blood loss, attenuate the inflammatory response, and lower complication rates in elderly patients undergoing surgery for intertrochanteric fractures.

## 1 Introduction

The increasing incidence of hip fractures among the elderly population, particularly those aged 60 years and older, poses significant challenges to healthcare systems worldwide. This population is especially vulnerable because of factors such as osteoporosis, age-related muscle loss, and an elevated risk of falls, which together contribute to substantial morbidity and mortality following hip fractures (Gálvez Márquez et al., 2025; Goru et al., 2025). Among the types of hip fractures, intertrochanteric fractures are especially common and often require surgical intervention to restore mobility and function (Meermans and van Egmond, 2025).

Recent studies have demonstrated that blood loss remains a critical concern during surgical procedures for hip fractures, significantly affecting patient outcomes (Lou et al., 2024). Tranexamic acid (TXA), an antifibrinolytic agent, has emerged as a promising strategy to limit intraoperative blood loss and reduce transfusion requirements (Fahmy et al., 2025; Majumdar et al., 2025). TXA acts by inhibiting fibrinolysis, thereby stabilizing formed clots and minimizing surgical bleeding (Barrett et al., 2025). Clinical trials have shown that TXA administration is associated with reduced blood loss and improved postoperative recovery across various surgical settings, including orthopedic procedures (McGarry et al., 2025; Triapthy et al., 2025; Zhao et al., 2025).

Despite the growing body of evidence supporting the use of TXA in intertrochanteric fracture surgeries, a gap remains in understanding its specific effects on inflammatory responses and long-term outcomes in elderly patients. Inflammatory markers such as interleukin-6 (IL-6) play a critical role in postoperative recovery and may influence complication rates and overall prognosis (Moldovan et al., 2023; Moldovan, 2024; Wang and Li, 2025). Elevated IL-6 levels have been linked to adverse outcomes after surgery, highlighting the need for interventions that effectively modulate perioperative inflammatory responses (García-Alvarez et al., 2024).

This study aimed to investigate the effects of intraoperative TXA administration on early postoperative inflammatory markers and clinical outcomes in elderly patients undergoing surgical fixation for intertrochanteric fractures. By analyzing serum IL-6 levels and other clinical outcomes, this study seeks to clarify the potential benefits of TXA beyond its hemostatic effects. These findings may contribute to refining surgical protocols and enhancing postoperative care strategies for this high-risk population.

## 2 Materials and methods

### 2.1 General information

Inclusion criteria: (1) diagnosis of intertrochanteric fracture caused by low-energy trauma and confirmed by X-ray or computed tomography; (2) age ≥ 60 years; (3) time from injury to surgery < 2 weeks; (4) availability of serum samples; (5) treatment with intramedullary nailing for fixation. Exclusion criteria: (1) presence of tumors, thyroid disease, or other endocrine disorders; (2) pathological fractures; (3) receipt of anti-osteoporosis treatment within 3 months before surgery or anti-inflammatory treatment within 1 week before surgery; (4) allergy to TXA; (5) history of or current thromboembolic events (e.g., deep vein thrombosis or pulmonary embolism); (6) concomitant injuries to major organs or fractures at other sites; (7) history of seizures, significant renal impairment (defined as a preoperative creatinine clearance <30 mL/min); (8) incomplete medical records.

**Figure 1:**
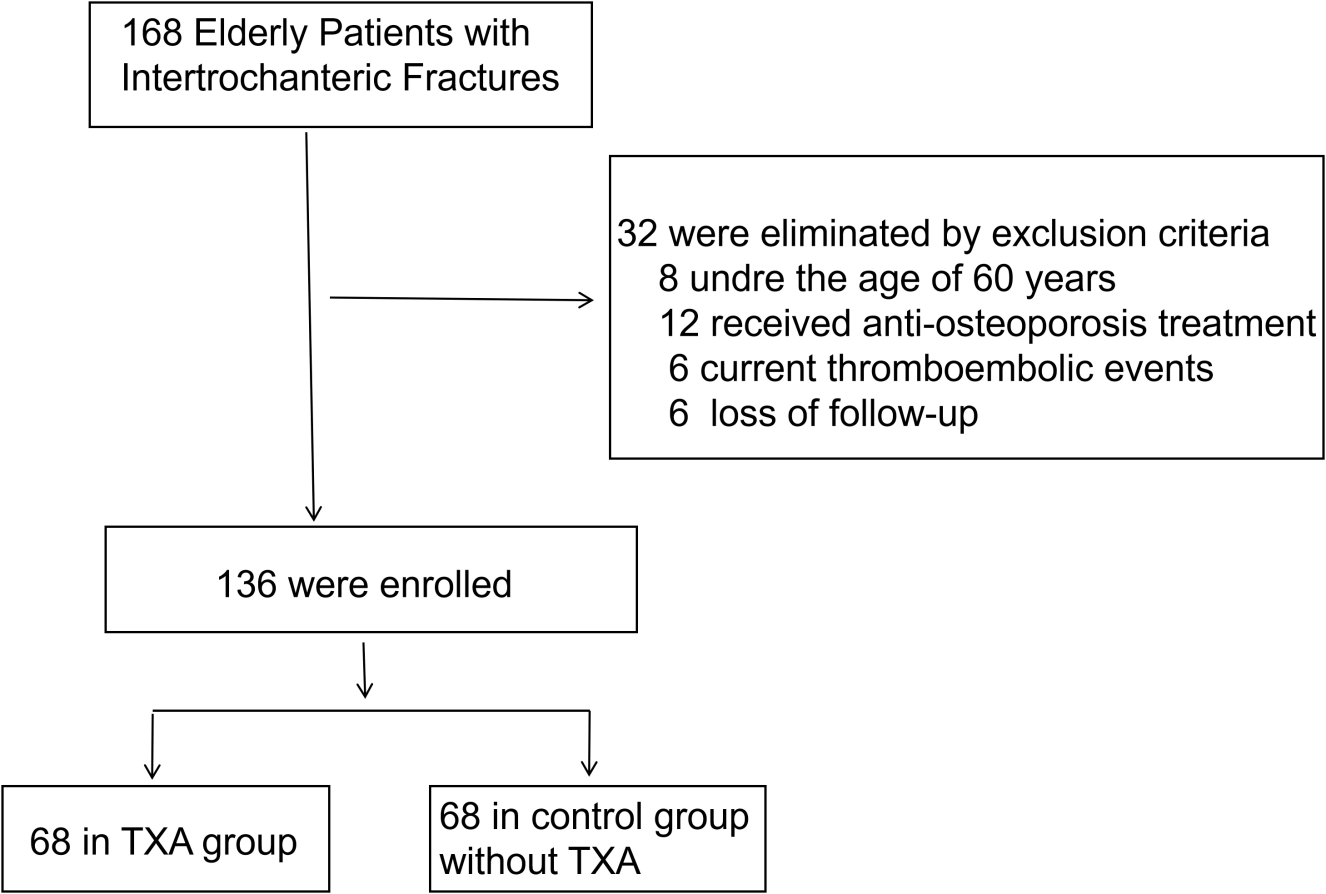
Flow diagram of included patients.

From January 2021 to December 2023, a total of 136 patients with intertrochanteric fractures met our inclusion and exclusion criteria (**Figure 1**) were enrolled from the Hospital of Chengdu University of Traditional Chinese Medicine, including 59 males and 77 females, aged 62–96 years (79.9 ± 8.4 years). All fractures were caused by low-energy trauma. The bone mineral density T-scores ranged from −5.4 to −0.1 SD (mean ± SD: −2.0 ± 1.0). All patients underwent proximal femoral intramedullary nailing. The decision to administer TXA was made by the attending anesthesiologist in consultation with the surgical team, considering the patient’s overall risk profile for bleeding and thrombosis. Sixty-eight patients received TXA during surgery [TXA group, intravenous drip (15 mg/kg)], whereas 68 patients did not (control group). No statistically significant differences were observed between groups in baseline characteristics (p > 0.05) (**Table 1**). Informed consent was obtained from all patients. This study was approved by the Hospital of Chengdu University of Traditional Chinese Medicine’s Clinical Research Ethics Committee (Approval No. 20250202).

**Table 1:** Baseline characteristics of patients.

Variables	TXA	Control	P
	(n=68)	(n=68)	
Gender			0.729
Male	31	28	
Female	37	40	
Age (岁, ± s)	79.1 ± 8.7	80.6 ± 8.1	0.299
smoking (n, %)	30(44.1)	28(41.1)	0.862
alcohol (n, %)	18(26.4)	20(29.4)	0.849
BMI (kg/m²)	22.8 ± 3.4	22.4 ± 3.1	0.474
Hypertension	35(51.4)	38(55.8)	0.731
Diabetes	20(29.4)	23(33.8)	0.848
Coronary heart disease	32(47.0)	30(44.1)	0.863
WBC (×109/L)	8.6 ± 3.1	8.5 ± 2.4	0.934
ALT[U/L, M (Q1, Q3)]	19.5(15.8, 25.5)	20.5(16.0, 28.3)	0.665
AST[U/L, M (Q1, Q3)]	27.0(21.0, 32.3)	28.0(23.8, 34.0)	0.376
BUN[mmol/L, M (Q1, Q3)]	6.9(5.4, 9.3)	7.2(6.2, 8.3)	0.574
Cr[μmol/L, M(Q1, Q3)]	76.3(61.5, 83.3)	72.5(63.8, 86.3)	0.362
TP(g/L)	75.3 ±8.1	73.5 ± 8.5	0.208
IL-6[pg/ml, M (Q1, Q3)]	12.5(8.9, 19.0)	13.5(9.0, 18.9)	0.676

### 2.2 Perioperative management

The surgical procedures and perioperative management were performed by the same team of experienced surgeons to ensure consistency and standardization of intraoperative and postoperative care. In the TXA group, TXA (15 mg/kg) was administered intravenously approximately 10 to 15 minutes before the skin incision to reduce intraoperative bleeding. No drainage tubes were placed postoperatively to minimize infection risk and facilitate early mobilization.

### 2.3 Follow-up and observation indicators

Patients were followed up regularly by telephone or during outpatient visits after discharge.For elderly patients (≥60 years) at our center, we routinely test for IL-6, α-TNF, and γ-IFN. If the results are abnormal, we will continue monitoring the patient. IL-6, operation duration, total blood loss (TBL), hidden blood loss (HBL), transfusion rate, incidence of deep vein thrombosis (DVT) in the lower limbs, and length of hospital stay were compared between the two groups. DVT was diagnosed based on doppler ultrasonography. All patients underwent doppler ultrasonography of thebilateral lower extremities before the surgery and after the surgery. Serum IL-6 levels and visual analog scale (VAS) scores were measured on postoperative days 1, 3, 5, and 7. The incidence of complications(pulmonary infection, cognitiveimpairment, cardio accident and cerebral accident) and mortality within one year after surgery were also recorded. Cognitive impairment was assessed by Montreal Cognitive Assessment (MoCA) score (Wong et al., 2022). Pulmonary infection was diagnosed based on clinical symptoms (e.g., cough, fever), radiographic evidence (chest X-ray or CT scan), and laboratory findings (e.g., leukocytosis, positive sputum culture). Data were extracted from inpatient medical records and follow-up visit notes. Cardiac events (including myocardial infarction, arrhythmia, and heart failure) were identified using electrocardiogram (ECG) changes, elevated cardiac biomarkers (troponin), echocardiography findings, and cardiology consultation notes. These events were captured from hospitalization records, emergency department visits, and routine follow-up assessments. Cerebral accidents were confirmed by neurologist evaluation and neuroimaging (CT or MRI).

### 2.4 Statistical analysis

All statistical analyses were performed using SPSS software (version 26.0; IBM Corp., Armonk, NY, USA) and GraphPad Prism (version 8.4.0; GraphPad Software, San Diego, CA, USA). The Shapiro–Wilk test was used to assess the normality of continuous variables. Normally distributed data were expressed as mean ± standard deviation, and intergroup comparisons were conducted using independent-samples t test. Non-normally distributed data were expressed as median (Q1, Q3), and between-group comparisons were performed using the Mann–Whitney U test. Categorical variables were expressed as counts and percentages, with between-group comparisons carried out using the χ² test. All statistical tests were two-tailed, and p < 0.05 was considered statistically significant.

## 3 Results

A total of 136 patients with intertrochanteric fractures were included, comprising 59 males and 77 females. Patients were followed for 12–24 months (mean ± SD: 14.9 ± 2.8 months) and were aged 62–96 years (mean ± SD: 79.9 ± 8.4 years). Demographic characteristics and preoperative blood test results showed no significant differences between the two groups (**Table 1**).

The mean operation time did not differ significantly between the groups (97.2 ± 14.3 min vs. 99.6 ± 12.2 min, p = 0.294) (**Table 2**). Mean TBL was significantly lower in the TXA group compared with the control group (981.74 ± 451.14 mL vs. 1206.27 ± 408.22 mL, *p* = 0.002). Similarly, mean HBL was significantly lower in the TXA group (890.94 ± 409.99 mL vs. 1104.39 ± 412.06 mL, p = 0.003). The blood transfusion rate was also significantly lower in the TXA group than in the control group (32.3% vs. 51.4%, p = 0.037). The incidence of DVT did not differ significantly between groups (36.7% vs. 48.8%, p = 0.860). The mean length of hospital stay was similar between groups (9.76 ± 4.62 days vs. 9.37 ± 4.16 days, p = 0.605) (**Table 2**).

**Table 2:** Surgical and perioperative outcomes of the two groups.

Variables	TXA	Control	P
	(n=68)	(n=68)	
Operation time(Min)	97.2 ± 14.3	99.6 ± 12.2	0.294
TBL(ml)	981.74 ± 451.14	1206.27 ± 408.22	0.002
HBL(ml)	890.94 ± 409.99	1104.39 ± 412.06	0.003
Transfusion rate (n, %)	22(32.3)	35(51.4)	0.037
DVT accidence (n, %)	25(36.7)	33(48.5)	0.860
Length of stay(day)	9.76 ± 4.62	9.37 ± 4.16	0.605

**Figure 2:**
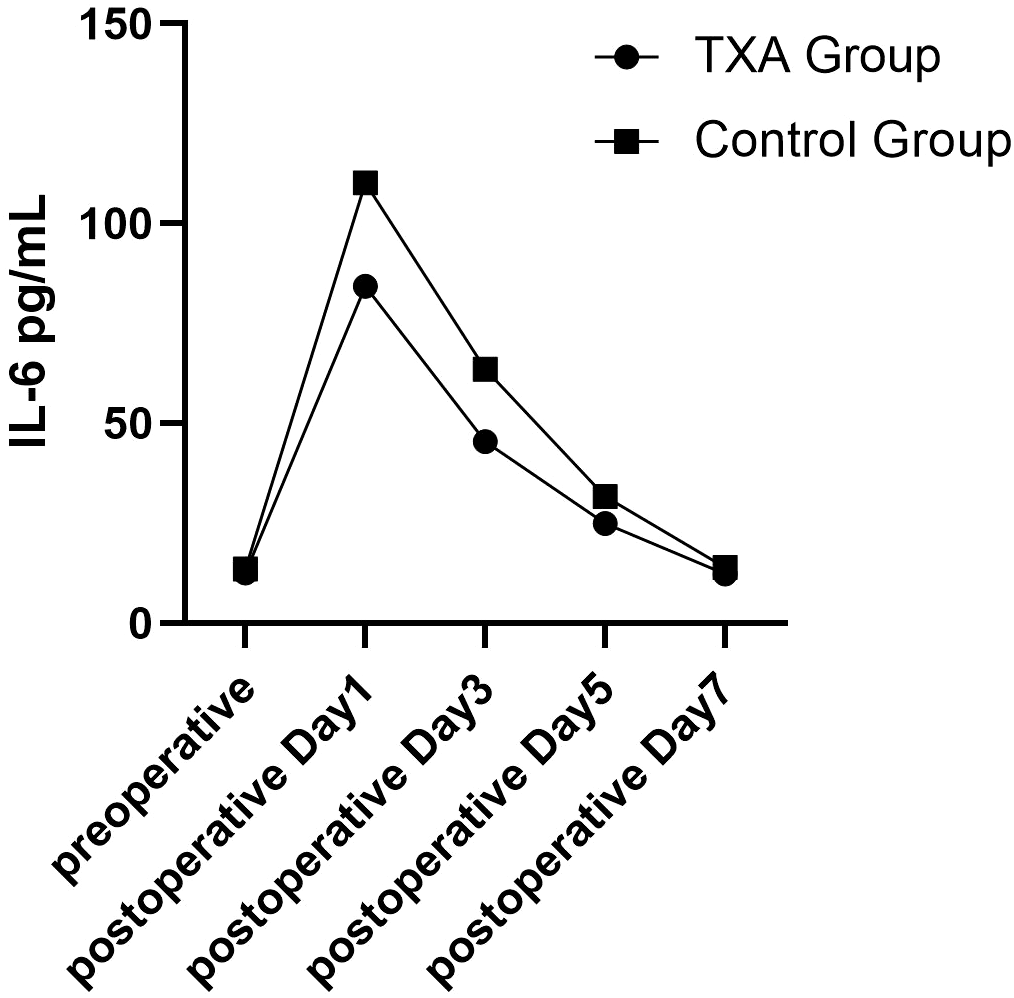
The trends in IL-6 levels over time.

On postoperative days 1, 3, and 5, serum IL-6 levels were significantly lower in the TXA group compared with the control group (p < 0.05). By postoperative day 7, no significant difference was observed between the groups (p > 0.05) (**Table 3**, **Figure 2**). VAS scores were significantly lower in the TXA group than in the control group on postoperative days 1 and 3 (p < 0.05). However, no significant differences were observed on postoperative days 5 and 7 (p > 0.05) (**Table 3**).

**Table 3:** Comparison of serum IL-6 levels and VAS scores between the two groups.

Variables	TXA	Control	P
	(n=68)	(n=68)	
IL-6			
Postoperative 1d	84.3(66.3, 100.1)	110.2(83.1, 162.9)	<0.001
Postoperative 3d	45.4(36.8, 61.2)	63.6(44.2, 84.2)	<0.001
Postoperative 5d	24.9(19.4, 37.5)	31.7(22.4, 42.9)	0.005
Postoperative 7d	12.3(9.3, 18.6)	13.9(9.3, 17.9)	0.788
VAS			
Postoperative 1d	4.5 ± 0.9	6.9 ± 1.2	<0.001
Postoperative 3d	2.4 ± 0.9	3.1 ± 1.2	<0.001
Postoperative 5d	1.8 ± 0.6	1.9 ± 0.7	0.372
Postoperative 7d	1.6 ± 0.4	1.7 ± 0.5	0.2

The incidence of complications within one year was significantly lower in the TXA group compared with the control group (26.4% vs. 48.5%, *p* = 0.032). One-year mortality was lower in the TXA group than in the control group (14.7% vs. 10.2%), although this difference was not statistically significant (*p* = 0.303) (**Table 4**).

**Table 4:** One-year complications and mortality in the two groups.

Variables	TXA	Control	P
	(n=68)	(n=68)	
complications(1year)(n, %)	18(26.4)	33(48.5)	0.032
pulmonary infection	6	6	
cognitiveimpairment	5	15	
cardio accident	3	7	
cerebral accident	4	5	
mortality(1year)	7(10.2)	10(14.7)	0.303

## 4 Discussion

Osteoporotic fractures, particularly intertrochanteric fractures, represent a significant public health concern among the elderly population, especially those aged 60 years and older. As the population continues to age, the incidence of these fractures is rising, correlating with higher morbidity and mortality rates. Intertrochanteric fractures are frequently caused by low-energy trauma, most often from falls, and can result in severe complications, including prolonged hospitalization, loss of independence, and increased mortality (Lu et al., 2022; Meermans and van Egmond, 2025). In older adults, factors such as low bone mineral density and comorbid conditions further increase the risk of sustaining such fractures (Chen et al., 2025; Meermans and van Egmond, 2025). Consequently, improving management strategies and exploring interventions that enhance surgical outcomes and reduce postoperative complications are of critical importance in this patient population.

In this study, we evaluated the effects of intraoperative TXA administration on early postoperative inflammatory markers and clinical outcomes in elderly patients undergoing surgical fixation for intertrochanteric fractures. A total of 136 patients were included, with 68 receiving TXA and 68 serving as controls. Our findings demonstrated that TXA significantly reduced total and hidden blood loss, decreased transfusion rates, and lowered postoperative IL-6 levels on days 1, 3, and 5. In addition, TXA use was associated with a lower complication rate within one year. These results indicate that TXA may be a valuable adjunct in the surgical management of intertrochanteric fractures, potentially contributing to improved recovery and reduced postoperative morbidity in this high-risk population.

Previous studies have primarily focused on the role of TXA in reducing blood loss and transfusion requirements across various surgical settings (Artykbay et al., 2025; Graif et al., 2025; Klingemann et al., 2025; Mutlu et al., 2025). Our results indicated a significant reduction TBL, HBL, and transfusion rates in patients who received TXA. No significant difference in DVT incidence was observed between groups. Importantly, this study specifically investigated TXA’s effects in elderly patients with intertrochanteric fractures, with particular emphasis on early postoperative inflammatory responses. Serum IL-6 levels were significantly lower in the TXA group than in the control group on postoperative days 1, 3, and 5, with no significant difference observed by day 7. TXA, as an antifibrinolytic agent, not only reduces intraoperative bleeding, which helps attenuate postoperative inflammatory responses, but also exerts direct anti-inflammatory effects through multiple pathways. TXA inhibits the binding of plasminogen to fibrin, thereby reducing plasmin generation, preventing excessive fibrinolysis, and indirectly decreasing the release of pro-inflammatory cytokines such as IL-6 and tumor necrosis factor alpha (Sugimoto et al., 2017; Barrett et al., 2019). In addition, plasmin activity is strongly linked to complement activation. By reducing plasmin activity, TXA suppresses complement components C3 and C5 activation, thereby reducing the production of complement C5a and mitigating complement-mediated inflammation (Amara et al., 2010; Leung and Morser, 2016). Barrett et al. reported that TXA may further reduce fibrinolysis-related inflammation by suppressing tissue-type plasminogen activator activity (Barrett et al., 2019). In elderly patients with intertrochanteric fractures caused by low-energy trauma, osteoporosis is common and contributes to increased intraoperative blood loss and a heightened inflammatory response after surgery (Wáng, 2024). These findings suggest that TXA may be particularly effective for elderly patients with intertrochanteric fractures and concurrent osteoporosis in reducing postoperative inflammation. However, further studies are warranted to elucidate the complex anti-inflammatory mechanisms of TXA and confirm these findings in larger, multicenter cohorts.

The significant reduction in overall complication rates within the first postoperative year among patients who received TXA further underscores the clinical importance of this study. The lower incidence of complications in the TXA group (26.4% vs. 48.5%) suggests that TXA may not only facilitate recovery but also contribute to improved long-term health outcomes in elderly patients undergoing surgery for femoral fractures. Sun et al (Sun et al., 2011) reported that excessive activation of inflammatory responses after surgery is strongly associated with a higher incidence of complications and increased mortality in older patients with hip fractures. Similarly, Rui et al (Rui et al., 2025) observed that patients with lower postoperative inflammatory markers following hip and knee arthroplasty had significantly shorter hospital stays. TXA has also been shown to reduce postoperative pain and decrease opioid consumption after total knee arthroplasty and anterior cruciate ligament reconstruction (Laoruengthana et al., 2019). This effect is likely mediated by its ability to reduce bleeding, minimize local swelling, and attenuate inflammation. Our findings are consistent with earlier reports by Cai et al (Cai et al., 2020) demonstrating the long-term efficacy of TXA in reducing postoperative complications, thereby supporting its clinical value in elderly fracture patients (Cai et al., 2020). These findings further advocate for the integration of TXA into standard surgical protocols for this population, with the potential to reduce healthcare costs and optimize resource allocation in postoperative care (Burns et al., 2025; Tejerina Álvarez et al., 2025). Although one-year mortality was lower in the TXA group compared with the control group (14.7% vs. 10.2%), the difference was not statistically significant (p = 0.303). These results are consistent with prior reports showing 1-year, 2-year, and 3-year mortality rates after intertrochanteric fracture surgery of 9.6%, 16.7%, and 24.4%, respectively (Lu et al., 2022). The incidence of DVT did not differ significantly between groups (36.7% vs. 48.8%, p = 0.860). However, the DVT incidence as this seems high. The incidence may appear high due to several factors, including our study’s rigorous and proactive screening protocol (routine Doppler ultrasound for all high-risk patients, not just symptomatic cases), the specific high-risk patient population enrolled (e.g., older age, major orthopedic surgery, cancer), and potential regional or institutional variations in patient risk profiles and thromboprophylaxis protocols (Lu et al., 2023; Cong et al., 2024).

This study has several limitations that warrant consideration. First, although the sample size was adequate for preliminary analysis, it may not fully capture the variability in patient responses to TXA across diverse demographic groups and comorbid conditions. Second, the retrospective design introduces potential selection and information biases, particularly in patient inclusion and outcome recording. The absence of a randomized controlled trial design limits the ability to draw definitive causal inferences regarding the efficacy of TXA. Third, although the follow-up period was sufficient to capture early postoperative outcomes, it may not adequately reflect long-term complications or functional recovery in this elderly population. Finally, the exclusive use of IL-6 as an inflammatory marker may overlook other relevant pathways and mediators involved in the postoperative inflammatory response.

## 5 Conclusion

In conclusion, the use of TXA in elderly patients undergoing surgical fixation for intertrochanteric fractures significantly reduced TBL, HBL, and early postoperative IL-6 levels, thereby improving short-term outcomes and lowering complication rates. These findings support TXA as a potentially valuable adjunct in the perioperative management of this patient population. However, further research—particularly large-scale, multicenter randomized controlled trials with extended follow-up—is needed to validate these results and to develop evidence-based guidelines for TXA use in geriatric orthopedic surgery.

## Data Availability

The original contributions presented in the study are included in the article/supplementary material. Further inquiries can be directed to the corresponding author.

## References

[B1] Amara U. Flierl M.A. Rittirsch D. Klos A. Chen H. Acker B. . (2010). Molecular intercommunication between the complement and coagulation systems. J. Immunol. 185, 5628–5636. doi: 10.4049/jimmunol.0903678. PMID: 20870944 PMC3123139

[B2] Artykbay S. Susantitaphong P. Tantavisut S. (2025). Efficacy and safety of topical tranexamic acid in elderly hip fractures undergoing surgical treatment: Meta-analysis of randomized controlled trials. Clin. Orthop. Surg. 17, 16–28. doi: 10.4055/cios24184. PMID: 39912079 PMC11791490

[B3] Barrett C.D. Moore H.B. Kong Y.W. Chapman M.P. Sriram G. Lim D. . (2019). Tranexamic acid mediates proinflammatory and anti-inflammatory signaling via complement C5a regulation in a plasminogen activator-dependent manner. J. Trauma Acute Care Surg. 86, 101–107. doi: 10.1097/TA.0000000000002092. PMID: 30575685

[B4] Barrett W.J. Kaucher K.A. Orpet R.E. Campion E.M. Goodloe J.M. Fischer P. . (2025). Tranexamic acid in trauma. Ann. Emerg. Med. 86, e73–e80. doi: 10.1016/j.annemergmed.2025.03.007. PMID: 40846436

[B5] Burns K. A. Robbins L. M. Humphrey L. LeMarr A. R. Morton D. J. Wilson M. L. (2025). Use of tranexamic acid reduces opioid consumption after arthroscopic rotator cuff repair. J. Shoulder Elbow Surg. 104, 757–764 doi: 10.1016/j.jse.2025.07.023. PMID: 40907869

[B6] Cai J. Ribkoff J. Olson S. Raghunathan V. Al-Samkari H. DeLoughery T.G. . (2020). The many roles of tranexamic acid: An overview of the clinical indications for TXA in medical and surgical patients. Eur. J. Haematol. 104, 79–87. doi: 10.1111/ejh.13348. PMID: 31729076 PMC7023891

[B7] Chen J. Gan X. Song J. Gao L. Shao M. Wang Y. . (2025). A systematic review and meta-analysis of barriers affecting early ambulation in older patients after hip fracture surgery. Nurs. Health Sci. 27, e70177. doi: 10.1111/nhs.70177. PMID: 40799003

[B8] Cong Y. Wang B. Fei C. Zhang H. Li Z. Zhu Y. . (2024). Dynamic observation and risk factors analysis of deep vein thrombosis after hip fracture. PloS One 19, e0304629. doi: 10.1371/journal.pone.0304629. PMID: 38829867 PMC11146713

[B9] Fahmy M. Karim M. A. Abdelazeem A. H. Abdelrazek A. M. (2025). Intravenous injection of tranexamic acid in patients with pelvic fractures: A prospective randomized trial. Hip Pelvis 37, 64–71. doi: 10.5371/hp.2025.37.1.64. PMID: 40012149 PMC11885788

[B10] Gálvez Márquez G. Muñoz Pascual A. Ojeda Thies C. García Cruz G. Sáez López M. P. Cordero Ampuero J. (2025). Can we improve early readmission after hip fracture of the adult? A retrospective analysis of 57.544 patients from SNHFR. Injury 56, 112680. doi: 10.1016/j.injury.2025.112680. PMID: 40848687

[B11] García-Alvarez F. Chueca-Marco Á. Martínez-Lostao L. Aso-Gonzalvo M. Estella-Nonay R. Albareda J. (2024). Serum levels of IL-6 and IL-10 on admission correlate with complications in elderly patients with hip fracture. Injury 55, 111736. doi: 10.1016/j.injury.2024.111736. PMID: 39068064

[B12] Goru V. Kumar S. Acharya K. Pai K. M. (2025). Survival at one year following surgery for intertrochanteric and femoral neck fractures in the elderly: A retrospective comparison. J. Clin. Orthop. Trauma 69, 103160. doi: 10.1016/j.jcot.2025.103160. PMID: 40838086 PMC12361797

[B13] Graif N. Warschawski Y. Amzallag N. Zarour S. Ben-Tov T. Snir N. . (2025). Effect of intraoperative tranexamic acid on blood loss and outcomes in intertrochanteric fractures: a retrospective study of 1728 patients. Eur. J. Orthop. Surg. Traumatol 35, 380. doi: 10.1007/s00590-025-04504-0. PMID: 40908344

[B14] Klingemann C. A. Lauritzen J. B. Jørgensen H. L. (2025). Efficacy and safety of tranexamic acid use on postoperative blood transfusion in hip fracture patients- a systematic review and meta-analysis. Eur. J. Trauma Emerg. Surg. 51, 164. doi: 10.1007/s00068-025-02846-2. PMID: 40192873

[B15] Laoruengthana A. Rattanaprichavej P. Rasamimongkol S. Galassi M. Weerakul S. Pongpirul K. (2019). Intra-articular tranexamic acid mitigates blood loss and morphine use after total knee arthroplasty. A randomized controlled trial. J. Arthroplast 34, 877–881. doi: 10.1016/j.arth.2019.01.030. PMID: 30755381

[B16] Leung L. L. Morser J. (2016). Plasmin as a complement C5 convertase. EBioMedicine 5, 20–21. doi: 10.1016/j.ebiom.2016.03.015. PMID: 27077104 PMC4816833

[B17] Lou L. Xu L. Wang X. Xia C. Dai J. Hu L. (2024). Comprehensive assessment of risk factors and development of novel predictive tools for perioperative hidden blood loss in intertrochanteric femoral fractures: a multivariate retrospective analysis. Eur. J. Med. Res. 29, 626. doi: 10.1186/s40001-024-02244-1. PMID: 39726041 PMC11670504

[B18] Lu Y. Huang Q. Xu Y. Ren C. Sun L. Dong W. . (2022). Predictors of long-term mortality after intertrochanteric fractures surgery: a 3-year retrospective study. BMC Musculoskelet. Disord. 23, 472. doi: 10.1186/s12891-022-05442-2. PMID: 35590357 PMC9118842

[B19] Lu D. X. Zhang K. Ma T. Li M. Li Z. Xu Y. B. . (2023). The association between admission serum phosphorus and preoperative deep venous thrombosis in geriatric hip fracture: A retrospective study. Diagnos (Basel) 13, 545. doi: 10.3390/diagnostics13030545. PMID: 36766651 PMC9914597

[B20] Majumdar P. K. Goyal G. Kumar V. Potalia R. Roy S. Punia P. (2025). Evaluation of efficacy of tranexamic acid on blood loss in surgically managed intertrochanteric fractures. J. Orthop. Case Rep. 15, 287–293. doi: 10.13107/jocr.2025.v15.i06.5740. PMID: 40520730 PMC12159644

[B21] McGarry L. Kearney J. Rotaru J. Gunaratne R. (2025). Swelling management in total knee arthroplasty: A systematic review. JBJS Rev. 13. doi: 10.2106/JBJS.RVW.25.00109. PMID: 40920881

[B22] Meermans G. van Egmond J. C. (2025). Malnutrition in older hip fracture patients: Prevalence, pathophysiology, clinical outcomes, and treatment-a systematic review. J. Clin. Med. 14, 5662 doi: 10.3390/jcm14165662. PMID: 40869488 PMC12386537

[B23] Moldovan F. (2024). Sterile inflammatory response and surgery-related trauma in elderly patients with subtrochanteric fractures. Biomedicines 12, 354. doi: 10.3390/biomedicines12020354. PMID: 38397956 PMC10887083

[B24] Moldovan F. Ivanescu A. D. Fodor P. Moldovan L. Bataga T. (2023). Correlation between inflammatory systemic biomarkers and surgical trauma in elderly patients with hip fractures. J. Clin. Med. 12, 5147. doi: 10.3390/jcm12155147. PMID: 37568549 PMC10419519

[B25] Mutlu T. Arıcan M. Karaduman Z. O. Turhan Y. Kaban İ. Dalaslan R. E. . (2025). Effect of oral + topical and only topical tranaxamic acid application on blood loss and postoperative transfusion in primary total hip arthroplasty. J. Clin. Med. 14, 1275. doi: 10.3390/jcm14041275. PMID: 40004805 PMC11856408

[B26] Rui C. Dai G. Tian C. Zhou S. Gao Y. Cao M. . (2025). Anti-inflammatory effect of multi-dose tranexamic acid in hip and knee arthroplasty: a systematic review and meta-analysis of randomized controlled trials. Inflammopharmacology 33, 917–928. doi: 10.1007/s10787-025-01679-0. PMID: 39992591

[B27] Sugimoto M.A. Ribeiro A. Costa B. Vago J.P. Lima K.M. Carneiro F.S. . (2017). Plasmin and plasminogen induce macrophage reprogramming and regulate key steps of inflammation resolution via annexin A1. Blood 129, 2896–2907. doi: 10.1182/blood-2016-09-742825. PMID: 28320709 PMC5445571

[B28] Sun T. Wang X. Liu Z. Chen X. Zhang J. (2011). Plasma concentrations of pro- and anti-inflammatory cytokines and outcome prediction in elderly hip fracture patients. Injury 42, 707–713. doi: 10.1016/j.injury.2011.01.010. PMID: 21349515

[B29] Tejerina Álvarez E. E. Cavada Carranza I. González Bermejo M. Molina García T. Lorente B.J.Á. (2025). Tranexamic acid applications in neurocritical patients: A narrative review. Med. Intensiva (Engl Ed) 49, 502139. doi: 10.1016/j.medine.2025.502139. PMID: 39890530

[B30] Triapthy S. K. Khan S. Varghese P. Patel H. Mishra N. Jain M. (2025). Safety and efficacy of tranexamic acid in pelvi-acetabular trauma surgery: a systematic review and meta-analysis of randomized controlled trials. Eur. J. Orthop. Surg. Traumatol 35, 387. doi: 10.1007/s00590-025-04506-y. PMID: 40928561

[B31] Wáng Y. (2024). For older women, the majority of hip fragility fractures and radiographic vertebral fragility fractures occur among the densitometrically osteoporotic population: a literature analysis. Quant Imaging Med. Surg. 14, 4202–4214. doi: 10.21037/qims-24-227. PMID: 38846307 PMC11151245

[B32] Wang Y. Li Y. (2025). Dexmedetomidine combined with propofol improves hemodynamic stability and recovery in elderly patients undergoing thoracoscopic lung cancer resection. Am. J. Transl. Res. 17, 4409–4420. doi: 10.62347/XCOK4904. PMID: 40672589 PMC12261165

[B33] Wong R.M.Y. Ng R.W.K. Chau W.W. Liu W.H. Chow S.K.H. Tso C.Y. . (2022). Montreal cognitive assessment (MoCA) is highly correlated with 1-year mortality in hip fracture patients. Osteoporos Int. 33, 2185–2192. doi: 10.1007/s00198-022-06426-7. PMID: 35763077

[B34] Zhao Z. Xing F. Luo W. Ma X. L. (2025). The optimal dose, efficacy and safety of tranexamic acid on hemorrhage control for high tibial osteotomy: A network meta-analysis. Orthop. Surg. 17, 2784–2808. doi: 10.1111/os.70140. PMID: 40936101 PMC12497536

